# PcExl1 a Novel Acid Expansin-Like Protein from the Plant Pathogen *Pectobacterium carotovorum*, Binds Cell Walls Differently to BsEXLX1

**DOI:** 10.1371/journal.pone.0095638

**Published:** 2014-04-22

**Authors:** Miguel Olarte-Lozano, Mario A. Mendoza-Nuñez, Nina Pastor, Lorenzo Segovia, Jorge Folch-Mallol, Claudia Martínez-Anaya

**Affiliations:** 1 Departamento de Ingeniería Celular y Biocatálisis, Instituto de Biotecnología, Universidad Nacional Autónoma de México, Cuernavaca, Morelos, México; 2 Centro de Investigación en Biotecnología, Universidad Autónoma del Estado de Morelos, Cuernavaca, Morelos, México; 3 Facultad de Ciencias, Universidad Autónoma del Estado de Morelos, Cuernavaca, Morelos, México; California State University Fullerton, United States of America

## Abstract

Microbial expansins act on plant cell walls similarly to plant expansins, albeit their loosening activity levels are tenfold lesser compared to plant expansins. We report the characterization of an expansin-like gene from the plant pathogen *Pectobacterium carotovorum*, named *exl*1. PcExl1 is an acidic protein that binds cellulose (Avicel), and weakens filter paper. The acidic nature of PcExl1 confers different binding properties when compared to *Bacillus subtilis* BsEXLX1, which is a basic protein. PcExl1 binding to wheat cell wall increased when acidic components were depleted, reaching a similar level to the binding to Avicel, indicating that cellulose is the target of PcExl1.

## Introduction

Plant cell walls are a network of complex polysaccharides including pectin, hemicellulose, and cellulose that provide structure and act as a barrier. Cell wall polymers are the structural components of the extracellular matrix, and a source of elicitors for plant defence responses to infection [1], [2]. Given the many physiological roles of this plant compartment, apoplastic pH, among other characteristics, is under tight control and kept within narrow limits [3]. Acidification of the apoplast induces plant growth through cell wall extension. Expansins are non-hydrolytic cell wall loosening proteins that produce cell wall extensibility under the turgor pressure of the cells, and are active at pH of 4.5 to 6 [4]. The molecular weights of expansin proteins are in the range of 25 kDa, and structurally they are composed of two compact domains (D1 and D2) that form an ellipsoid. Sequences with low sequence similarity to plant expansins, but highly similar at the structural level, have been found in other organisms such as fungi, bacteria and nematodes [5]. Besides the canonical expansin protein module, bacterial expansins can contain extra domains such as carbohydrate binding domains (CBMs) and cellulases [6]. On the other hand, proteins with structural similarity to D1, and lacking a D2, such as cerato platanin from *Ceratocystis platani* [7] and loosenin from *Bjerkandera adusta* [8], have been reported to bind polysaccharides and act on cellulose. The best characterised bacterial expansin to this date is BsEXLX1 from *Bacillus subtilis* [9], [10]. Structural, biochemical and biophysical analyses [9]–[12] indicate that binding of BsEXLX1 to components of the cell wall depends on electrostatic and hydrophobic interactions. Recent analyses of substrate binding *in muro* determined that BsEXLX1 binds the cellulose microfibrils, with cell wall loosening consequent to this interaction [12]. Binding to the hydrophobic pyranose rings in cellulose occurs with the open surface formed by a planar arrangement of three aromatic residues (W125/W126/Y157) in D2, similar to type A CBMs with affinity to crystalline cellulose [9]. For this reason, family CBM63 of the Carbohydrate-Active Enzymes database (CAZY) now groups together similar D2 domains of microbial expansins [10]. In contrast, conserved residues in D1 that form a shallow groove that is considered the putative polysaccharide binding surface (PPBS) are mainly polar, suggesting an interaction with the hydroxyl groups of the sugar rings [9]. Electrostatic forces outside of the PPBS are important for expansins to bind polysaccharides other than cellulose. BsEXLX1 is a basic protein, and salt interferes with binding of BsEXLX1 to negatively-charged groups of hemicellulose and pectins, decreasing more than 90% of the protein binding to intact cell walls at 10 mM CaCl_2_ [10]. The exact biophysical mechanism of cell wall loosening of expansins is not known, but it depends on the protein binding to cellulose, as mutants that decrease binding to other polysaccharides but not to cellulose retain their loosening ability, but not the other way around [10].

Many of the putative homologues of bacterial expansin genes are found in species that interact with plants, either as beneficial endophytes, soil saprophytes or pathogens [5]. Evidence for the involvement of expansin-like proteins during infection has come from the nematode *Globodera rostochiensis* that expresses a functional expansin that is secreted simultaneously to glycanase enzymes [13]. Also, a mutant BsEXLX1^–^ strain is greatly reduced in the ability of *B. subtilis* to colonize maize roots, however, the physiological role of expansin activity in bacteria (*e.g.*, during infection) has yet to be determined. Compared to the activity shown by plant expansins, prokaryotic expansins have tenfold lesser specific activity on vegetal tissues, and to overcome either weak or variable results, a brief alkali-pretreatment of the samples has been necessary [6], [10]. Besides binding to polysaccharides, BsEXLX1 has high affinity for peptidoglycan, suggesting that it could serve as a bifunctional agent localizing the bacterial cell within the plant cell wall [9]. Finally, expansins are attractive proteins for potential biotechnological applications, specifically for the production of biofuels from lignocellulose. On this respect, there are contradicting results in the literature on the existence of synergism between bacterial expansins and cellulases or xylanases [6], [14].

Here we report the characterization of the expansin-like protein PcExl1 from the plant pathogen *Pectobacterium carotovorum* (formerly *Erwinia carotovora*). Weakening of filter papers strips indicates expansin activity on cellulose that depends on conserved residue Asp82, and the aromatic triad Y125/W126/Y157. The acidic nature of PcExl1, compared to BsEXLX1 from *Bacillus subtilis*, has consequences for its affinity for cell walls of different plant species. This difference could be an important factor for the range of infectivity shown towards different crops for this organism.

## Experimental Procedures

### Cloning and Purification of PcExl1

Genomic DNA was extracted from the environmental strain *P. carotovorum* 101 [15], and PcExl1 was amplified using the following oligonucleotides: *Sfi*PcaF 5′-aactaggcccagccggccatggctgcccagtgggaactggac-3′ (thus creating a fusion to the PelB signal peptide to target the chimera to the periplasm), and *Not*PcaR1∶5′-tcgatgcggccgcttaaagctggacgttacccgt-3′ that includes a stop codon. This fragment excluded the predicted signal peptide determined with Signal P 4.1 Server [16]. Gene mutagenesis of residues D82 and Y125/W126/Y157 for Ala was performed using the QuickChange Lighting Multi Site-Directed Mutagenesis Kit (Agilent Technologies) following the manufacturer’s indications. Mutagenic oligonucleotides were: 5′-gactgtgcattggctttatcgtttaacg-3′, 5′-gttcaaaccctgcggcggctgcggtgcaat-3′ and 5′-cagaaaaccgatgctaaccactttatt-3′. The 661 bp product was digested with endonucleases *Sfi*I and *Not*I, and cloned into plasmid pET22b-S/N. Oligonucleotides synthesis and gene sequencing were performed by the Unidad de Síntesis y Secuenciación de DNA (IBT-UNAM). *Escherichia coli* BL21–DE3 were transformed, and expression was induced in cultures grown to OD_600_ 0.9 in LB medium at 37°C, at which point IPTG was added to a final concentration of 1 mM; further incubation was carried out at 16°C for 12 h. Periplasmic proteins were obtained by osmotic shock by incubating 15 min in 10% of the culture volume of buffer SET (20% sucrose, 5 mM EDTA and 20 mM TRIS) and centrifugation at 4°C, 15 min, 8200×*g.* The pellet was then incubated in the same volume of 5 mM MgSO4, and centrifuged under the same conditions. PcExl1 (WT and mutants) purification was carried out in a DEAE chromatography column, eluting with a gradient of 0 to 0.5 M NaCl and 20 mM sodium phosphate pH 8.0. Desalting was performed by ultrafiltration with 10 kDa cutoff membranes (Vivaspin, GE Healthcare) and 20 mM sodium phosphate pH 7.5. BsEXLX1-6×His was purified in a NTA-Ni2+ column (HisTrap, GE Healthcare), eluting with 20 mM phosphates buffer pH 7.5, 0.5 M NaCl and 250 mM imidazole. Desalting was carried out as explained before. Protein was quantified with the Bradford dye-binding method using the BioRad Protein Assay reagent following the manufacturer’s indications. Proteins were visualised in 15% SDS-PAGE gels containing 0.5% of 2,2,2-trichloroethanol [17], using a Stain-Free Tray in a Gel Doc EZ photo documentation system (BioRad).

### Differential Scanning Calorimetry

It was performed with 450 µl of pure preparations of PcExl1at 0.4 mg/ml in 20 mM HEPES buffer pH 7.5. PcExl1 thermograms were obtained from 25 to 90°C or from 35 to 65°C, at a rate of 90 K/h (in a Differential Scanning Calorimeter Cap-DSC equipment), to determine the Tm and the reversibility of the denaturation process, respectively. Comparisons to buffer, 20 mM HEPES pH 7.5, were included.

### Plant Materials

Seeds from wheat, cucumber and common bean were germinated and grown for 4 to 5 days (until seedlings were 5 cm in length) in a dark and humid tray at 25°C. Coleoptiles were cut at the base and abraded with pumice powder to remove the cuticle allowing the rapid penetration of buffers and proteins into the cell walls [18]. Samples were frozen in dry ice and kept at −70°C until use. Onion and celery were bought from a local supermarket. To obtain total cell walls (fraction 1), tissues were pulverized in dry ice (cucumber, wheat and bean coleoptiles), or blended with distilled H_2_O (onion and celery), then washed 5 times (2 h each) with 1 M NaCl and gentle stirring at 4°C. Sequential extraction of polysaccharides was performed by incubation in 50 mM CDTA in 20 mM potassium phosphate pH 7.0 to remove pectins (fraction 2), and then 4 M KOH, 0.5% NaBH_4_ to eliminate hemicelluloses (fraction 3). The remaining fraction was washed with H_2_O and dried at room temperature for three to four days.

### Binding Assays

Expansins PcExl1 or BsEXLX1 (5 µg) were incubated with 1 mg of insoluble substrate (cell wall, filter paper or Avicel) in 50 µl of buffer (20 mM acetate buffer ph 3.5; 20 mM citrate buffer pH 5; 20 mM HEPES buffer pH 7.5; and 20 mM borate buffer pH 9), for 1 h at 25°C, and agitated at 1000 rpm in a Thermomixer incubator (Eppendorf). After incubation samples were centrifuged for 10 min in a bench microcentrifuge at maximum speed at room temperature, 20 µl of the supernatant were mixed with sample buffer and loaded in 15% SDS-PAGE gels (as mentioned before). The amount of protein bound to the substrates was determined by the difference in signal obtained from a loading control and the remnant of the treated samples determined by densitometry with Image Lab 3.0 software (Bio-Rad). Results are expressed as µg bound protein/mg substrate. To determine the binding parameters *K_d_* and *B_max_* to Avicel, a depletion isotherm was obtained by incubating increasing amounts of protein (from 1 to 7 µM) with 5 mg of Avicel. Data was fitted to a single site Langmuir isotherm with GraphPad Prism software version 5.01.

### Tension Measurements of Filter Paper Strips

Filter paper (Whatman no. 1) weakening was determined as follows. Strips of 38 mm×5 mm were incubated in 3 ml of buffer alone, or with 120 µg/ml of protein during 1 h at room temperature and gentle rotation, then breaking force was determined with a Universal Testing Machine (Instron 5500R, USA), set up at 1 mm/min crosshead speed. Statistical significances were determined by Fisher’s LSD test for the different treatments (*p*<0.05).

### Cellulase and Xylanase Activities

Substrates (2.5 mg xylan from beechwood or Avicel, both from Sigma) were incubated with 100 µg/g substrate of endo-1,4 xylanase from *Trichoderma longibrachiatum* (Sigma) or 0.25 U cellulase from *Trichoderma reesei* (Sigma), respectively, in 20 mM HEPES buffer pH 7.5 only or added with up to 150 µg/ml of PcExl1 or BSA. Reactions were carried out in 600 µl at 30°C and agitated at 600 rpm in a Thermomixer incubator (Eppendorf). Reducing sugars concentration was determined by the 3,5-dinitrosalicylic acid method [19] and compared to D-xylose or D-glucose standard curves.

### Statistical Analysis

Groups of data were statistically compared by an ANOVA with *p*<0.05. Comparisons among individual means were made by Fisher’s least significant difference (LSD) *post hoc* test after ANOVA.

## Results

### Cloning and Expression of *Pectobacterium Carotovorum* PcExl1

**Figure 1:**
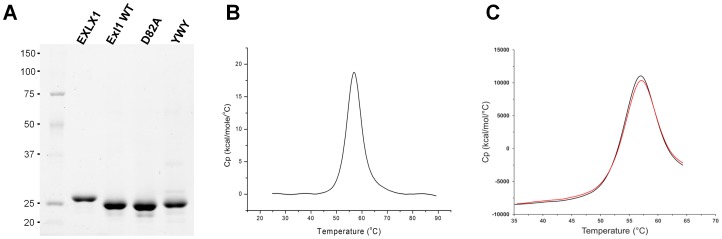
PcExl1 heterologous expression in *E. coli* produces a structured protein. (a) SDS-PAGE gel of purified *P. carotovorum* PcExl1 wild-type (WT), and mutants: D82A and Y126A/W127A/158Y (YWY), and comparison to purified *B. subtilis* BsEXLX1. PcExl1 WT and mutant variants are ∼22.9 kDa, whereas BsEXLX1 is ∼24.2 kDa. (b) Thermal denaturation of a solution of 0.4 mg/ml PcExl1, carried out at 90 K/h, showed a two-state process that occurs at 56.8°C. (c). PcExl1 denaturation is reversible after heating at 65°C (red line), under the same heating conditions as in (b).

*Pectobacterium carotovorum* strain 101 is an environmental strain originally isolated from an infected *Opuntia* [15]. Genomic DNA from this strain was used as a template to amplify a 661 bp sequence with homology to bacterial expansins. The oligonucleotide sequences were based on the available genome sequence of strain PCC21. We called this gene *exl*1, coding for expansin-like protein 1. Because the protein acts outside the cell, it contains a predicted secretion signal peptide with a cutting site between A23 and Q24. The forward oligonucleotide sequence was designed to initiate amplification of the protein at Ala23 in order to exclude the native signal peptide, while maintaining the Pro-Ala-Met-Ala cleavage site of the PelB signal peptide, to which PcExl1 was fused. The reverse primer included the PcExl1 native stop codon. For practical comparison to BsEXLX1, we have used amino acids numbering of PcExl1 based on that of BsEXLX1, that is, W25 in the unprocessed peptide was considered W1 in the mature protein. Sequencing of the PcExl1 clone (GenBank accession number KF988136) showed differences at the nucleotide level when compared to strains PCC21 (PCC21_021360) and PC1 (PC1_2092) resulting in 96.8 and 90.2% identity, respectively. However, the predicted protein is 100% identical to that of strain PCC21, whereas in PC1 a change of Pro for Ala was found in residue 151 in PcExl1 and PCC21. Recombinant PcExl1 protein was abundantly expressed in soluble form in the periplasm of *E. coli*, from where it was purified. Compared in a SDS-PAGE gel, PcExl1 has a faster electrophoretic mobility than BsEXLX1 from *Bacillus subtilis* (Figure 1a). Differential scanning calorimetry of pure preparations of PcExl1 indicated a denaturation Tm of 56.8°C (Figure 1b), with a denaturation profile similar to that of BsEXLX1 as determined by Kim et al. [20]. Re-folding of PcExl1 from the complete unfolded state at 90°C does not occur (not shown), however elevating the temperature to just above the denaturation peak (65°C) for a second time showed protein re-folding (Figure 1c), and therefore the denaturation process is reversible.

### Expansin Activity of PcExl1

**Figure 2:**
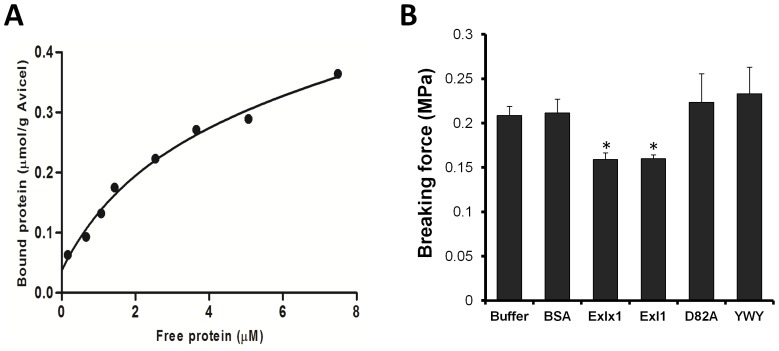
PcExl1 binds and acts on cellulose. (a) Binding isotherm of PcExl1 to Avicel. The result shown is representative of two more determinations. (b) Weakening activity of filter paper by PcExl1. Filter paper strips were incubated for one hour in phosphate buffer pH 7.5 alone or with 120 µg/ml of PcExl1 or BsEXLX1 or bovine serum albumin (BSA) for one hour, and then immediately analyzed in a Universal Testing Machine. Experiments are the result of at least six determinations. *Significant differences (*p*<0.05, Fisher’s LSD test).

Because expansins act on cellulose [12], we determined the binding parameters of PcExl1 to Avicel (microcrystalline cellulose) by Langmuir isotherms (Figure 2a). Binding parameters were *B_max_* 0.33 µmol/g Avicel, and *K_d_* 2.97 µM, almost identical to the reported parameters found for BsEXLX1 [10]. Treatment of filter paper strips with bacterial expansins decreases their breaking force under tension [10], [14]. To determine whether PcExl1 produces the same effect, filter paper strips were incubated in the presence of the protein for one hour and the tensile force required to break them was measured. PcExl1 provoked significant filter paper weakening, as the force required to break the strip was reduced from ∼0.20 MPa when the sample was incubated with buffer or BSA, to 0.16 MPa (Figure 2b). The same level of reduction was also induced by BsEXLX1, and is in agreement with previous reports in the literature [10]. It has been reported that a number of conserved residues in D1 of BsEXLX1: D71, Y73 and D82 are important for activity, whereas in D2 the mutation of the aromatic residues W125, W126 and Y152 for Ala also eliminates activity [10]. To determine if these residues are also important in PcExl1 two mutants were created: D82A in D1 and the triple aromatic Y125A/W126A/Y157A mutant (YWY) in D2 (Figure 1a). Both mutants showed reduced activity, as in each case weakening of the filter paper was abolished (Figure 2b), indicating that D82 and the three conserved aromatic residues in D2 in PcExl1 are also important for activity on filter paper. All these results confirm that PcExl1 acts on cellulose, and therefore PcExl1 can also be considered a functional bacterial expansin. Binding and activity levels of both PcExl1 and BsEXLX1 on Avicel and filter paper respectively, suggest that the PPBS is similar in both proteins.

### PcExl1 Binding to Substrates of Different Plant Species

**Figure 3:**
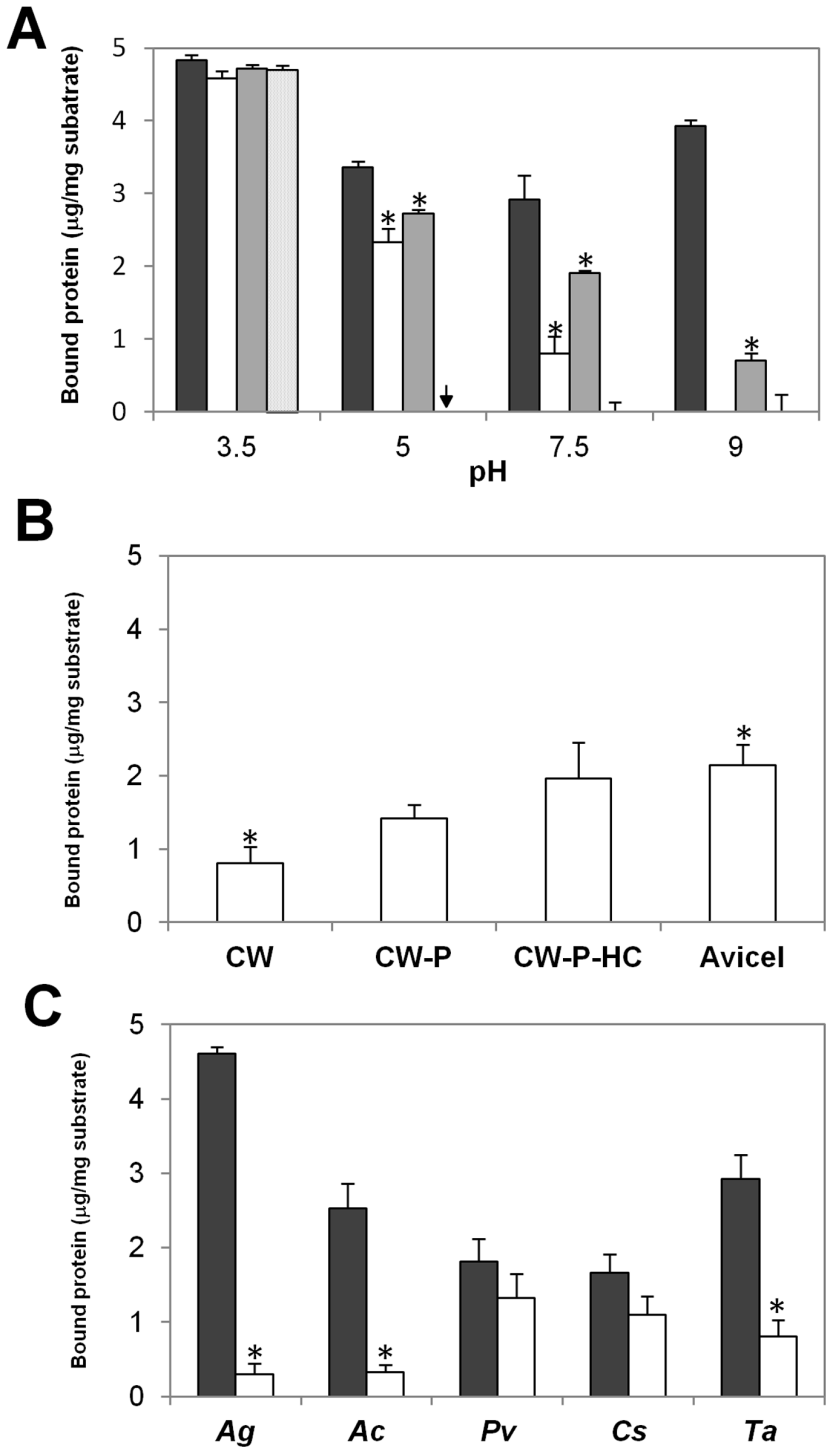
PcExl1 and BsEXLX1 bind differently to whole plant cell walls. (a) pH influence on binding of expansins (BsEXLX1, dark grey; PcExl1, white; D82A, black; YWY, dotted) to wheat coleoptile cell walls. The arrow indicates that YWY mutant was unstable at pH 5 in citrate buffer, and only a protein smear was detected, impeding quantification. *Significant differences at the different pHs (*p*<0.01, Fisher’s LSD test). (b) Binding of PcExl1 to wheat coleoptiles whole cell walls (CW), or cell wall fractions after extraction of polysaccharides: pectin-depleted (CW-P), and pectin- and hemicellulose-depleted (CW-P-HC), or Avicel. *Significant differences (*p*<0.05, Fisher’s LSD test). (c) Binding of expansins (BsEXLX1, dark grey; PcExl1, white) to cell walls from different plant species: *Apium greveolens* (*Ag*), *Allium cepa* (*Ac*), *Phaseolus vulgaris* (*Pv*), *Cucumis sativus* (*Cs*), and *Triticum aestivum* (*Ta*). In all cases, 5 µg of protein were incubated with 1 mg of substrate for one hour at 1000 rpm and 25°C. Results are the arithmetic mean and standard error of at least three independent experiments. ^*^Significant differences between PcExl1 and BsEXLX1 binding to the same plant species (*p*<0.05, Fisher’s LSD test).

After confirming that PcExl1 binds to cellulose, we aimed to determine PcExl1 binding to whole cell walls from wheat coleoptiles. After 1 h of incubation at pH 7.5, PcExl1 binding to wheat coleoptiles was significantly lesser than BsEXLX1 binding (0.8 µg/mg substrate *vs.* ∼2.9 µg/mg substrate) (Figure 3a). The amount of protein bound to the substrate was dependent on the pH of the incubation buffer, and it was higher for PcExl1 in more acid conditions, increasing to 2.3 µg/mg substrate at pH 5 (which was still significantly less compared to BsEXLX1), and decreasing to undetectable levels at pH 9. On the other hand, BsEXLX1 binding to wheat coleoptiles presented less variation, with a maximum binding capacity in this condition at pH 3.5 (Figure 3a). The capacity of PcExl1 to bind to wheat coleoptiles was significantly increased by the D82A mutation, as at pH 7.5 it was twofold greater than the wild type, and it restored the binding capacity at alkaline pH (0.65 µg/mg substrate). Finally, the YWY mutant completely abolished binding to wheat coleoptiles at pHs 7.5 and 9, while at acidic conditions binding reached 4.7 µg/mg substrate, which is the same level bound by all other proteins. These results suggest that at pH 3.5 protein binding is unspecific as there were no significant differences between the different proteins, whereas differences between expansins arise at more physiologically relevant conditions. Surprisingly, mutant WYW was unstable at pH 5 in citrate buffer, as it seemed to degrade, impeding protein quantification.

Given that PcExl1 binds to Avicel, we were interested to determine its binding to wheat coleoptiles after extracting the pectins and the hemicellulose from the cell wall. Pectin removal increased PcExl1 binding by 12% with respect to non-depleted cell walls (0.8 µg/mg substrate *vs.* 1.41 µg/mg substrate, taking 5 µg of protein as the maximum possible value), while pectin and hemicellulose depletion resulted in a further 11% increment with respect to complete cell walls (0.8 µg/mg substrate *vs.* 1.9 µg/mg substrate). The remaining cell wall was bound by PcExl1 at the same level as Avicel, to approximately 40% (Figure 3b). These results indicate that in the cell wall, cellulose is the binding site for Exl1. *Pectobacterium carotovorum* strains infect a broad range of plant species of economic interest. We determined the infectious capacity of strain 101 in different monocot and dicot plant species: *Apium greveolens* (*Ag*, celery), *Allium cepa* (*Ac*, onion), *Phaseolus vulgaris* (*Pv*, common bean), *Cucumis sativus* (*Cs*, cucumber), and *Triticum aestivum* (*Ta*, wheat). Dicot species (*Pv*, *Cs* and *Ag*) were susceptible to infection whereas monocots (*Ac* and *Ts*) did not show symptoms at 48 h post inoculation and therefore were considered resistant to infection. Experiments at pH 7.5, using cell walls from these plants showed variable levels of binding for both expansins. BsEXLX1 presented greater affinity to cell walls from most vegetables than PcExl1 (Figure 3c). Affinity of BsEXLX1 was *Ag>Ta = Ac>Pv = Cs*, whereas for PcExl1 was *Pv = Cs>Ta = Ag = Cs*; however, maximum binding was just above 25% with respect to the total protein in the reaction (5 µg). Comparing the two expansins, the maximum difference was 16-fold greater binding of BsEXLX1 to celery over PcExl1, and the minimum difference was for bean and cucumber being approximately 1.5-fold greater for BsEXLX1 (Figure 3c). The order of preference of BsEXLX1 over PcExl1 was *Ag>Ac>Ta>Cs = Pv*. Different binding levels between expansins, and between the same expansin to the various plants cell walls, could be a reflection of the acid content in these cell walls. The more acidic the cell wall, the larger the difference between the binding of the two proteins.

### Electrostatic Comparison between BsEXLX1 and PcExl1

**Figure 4:**
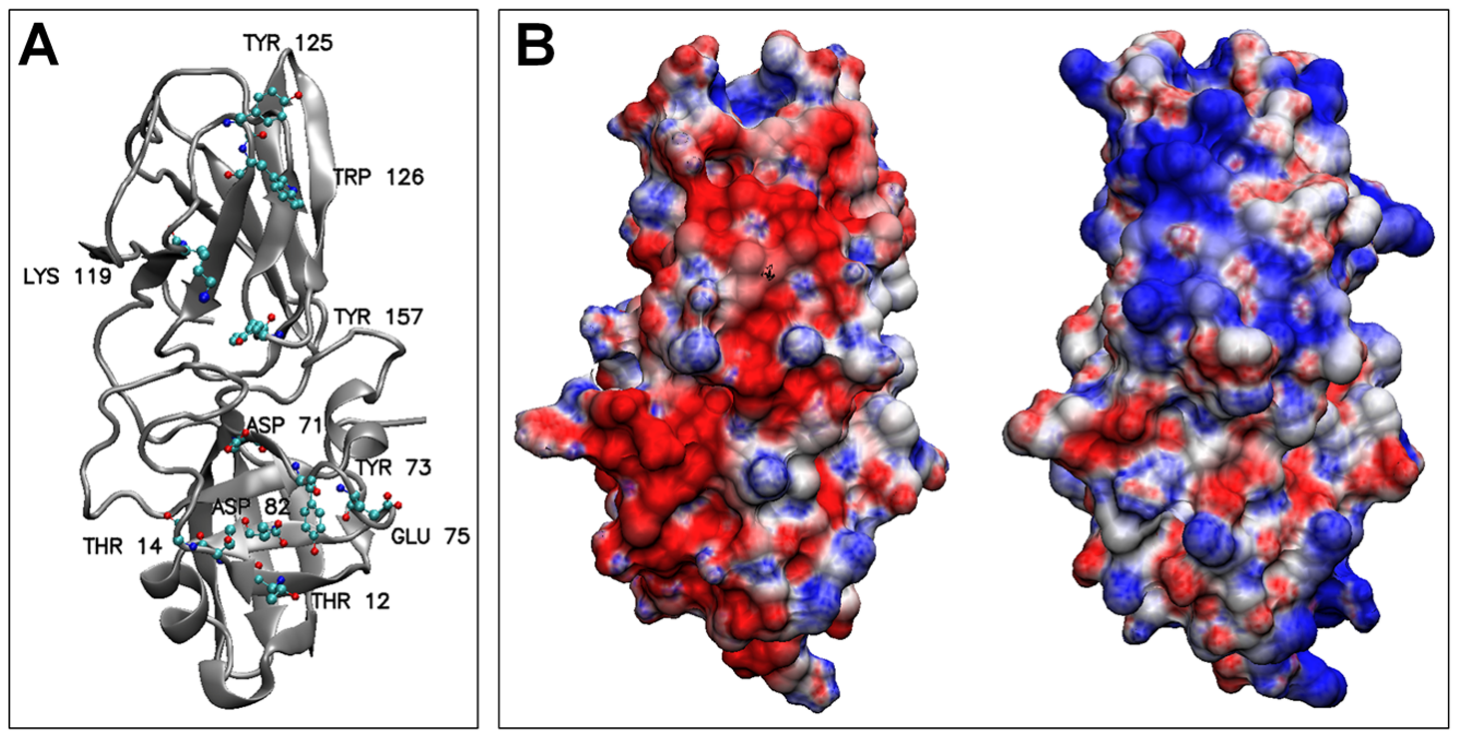
PcExl1 is an acidic protein with a similar structure to BsEXLX1. (a) Predicted carbohydrate binding surface of PcExl1. The backbone of the protein is depicted by a silver ribbon, and the sugar-binding residues are shown as labeled balls and sticks. (b) Electrostatic potential mapped at the protein surface, for the “back” of PcExl1 (left) and BsEXLX1 (right). Positive potential is shown in blue and negative potential in red, for a range of −5 to +5kT/e. The proteins are rotated 180 degrees with respect to panel (a), with the carbohydrate binding domain D2 at the top, and the double-psi beta barrel D1 at the bottom.

**Figure 5:**
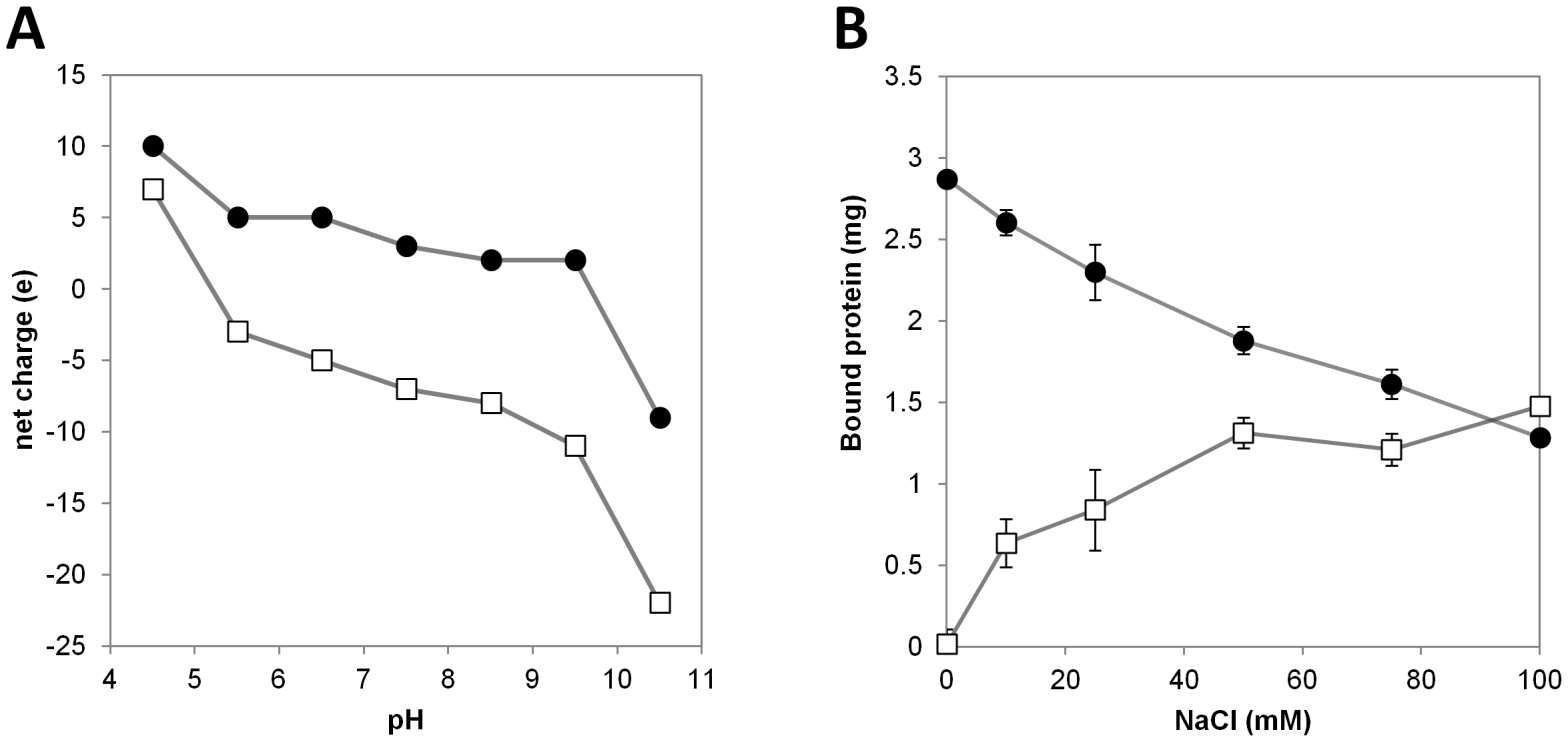
Expansins net charge depends on pH, and ionic strength determines their interaction with cellulose. (a) Theoretical net charge of the proteins at different pH values: full circles correspond to BsEXLX1 and open squares to PcExl1. (b) Ionic strength effect on binding to Avicel was determined experimentally, by incubating 1 mg of substrate with 5 µg of expansins (BsEXLX1, full circles; PcExl1, open squares) for one hour. Results are the arithmetic mean and standard deviations of three independent experiments.

PcExl1 is 56.5% identical to BsEXLX1, and all the critical residues for cellulose binding identified in BsEXLX1 [10] are either conserved or substituted for similar residues (W125 in BsEXLX1 and Y125 in PcExl1). An important difference is found in the high number of positively charged residues (R+K) in BsEXLX1 compared to PcExl1 (27 *vs*. 16, respectively). This difference is the reason that PcExl1 is an acidic protein with a predicted pI of 4.8, whereas BsEXLX1 has a pI >9.0, as noted by others [9]. A 3D model for PcExl1, starting at W1, was obtained from I-TASSER [21], with a C-score of 1.82 and a TM-score of 0.97±0.05. These scores reflect high confidence in the proposed model, and support the role of PcExl1 as a functional expansin. The templates used by I-TASSER were the crystal structures of BsEXLX1 (PDB 3D30 and 2BH0), and the recently deposited structure of an expansin from *Clavibacter michiganensis* (PDB 4JCW). Superposition of the model and the crystal structure of BsEXLX1 (PDB 3D30) reveals the equivalent positions of conserved residues in the PPBS (Figure 4a), as expected from the functional analysis presented above. Analysis of the electrostatic profiles of the two proteins shows that important differences between them exist outside the PPBS, which is electrostatically similar. These were calculated with the APBS server [22], assigning PARSE charges with PDB2PQR [23], and performing the calculation without added salt with the default parameters in the server. Taking the PPBS as the “front” of the molecules, the “back” is the region where most of the charge reversals between BsEXLX1 and PcExl1 are located. At a pH of 7.5, this leads to a positive surface for BsEXLX1, and a negative surface for PcExl1 (Figure 4b), offering a rationale for the pH dependence of cell wall binding by the two proteins (Figure 4a). Furthermore, calculations of net charge with PROPKA [24], [25], at pH ranging from 4.5 to 10.5 for both proteins, indicate that BsEXLX1 has a mildly positive charge (+5 to +2e) at a functional pH range [10], while PcExl1 has negative net charge (−3 to −11e) at the same pH range (Figure 5a). This persistent negative charge could explain the poor binding to negatively-charged cell walls, and the improvement in binding seen upon removal of pectin and hemicellulose (Figure 3b). The effect of NaCl in binding to Avicel was determined for both proteins. When no salt was added in the incubation buffer, BsEXLX1 exhibited maximum binding to the substrate at 2.9 µg/mg Avicel, and it decreased 2.2-fold as NaCl concentration increased (Figure 5b). The opposite effect was observed for PcExl1, which binds Avicel more avidly at high salt concentrations (Figure 5b).

### PcExl1 and Glycoside Hydrolases

**Figure 6:**
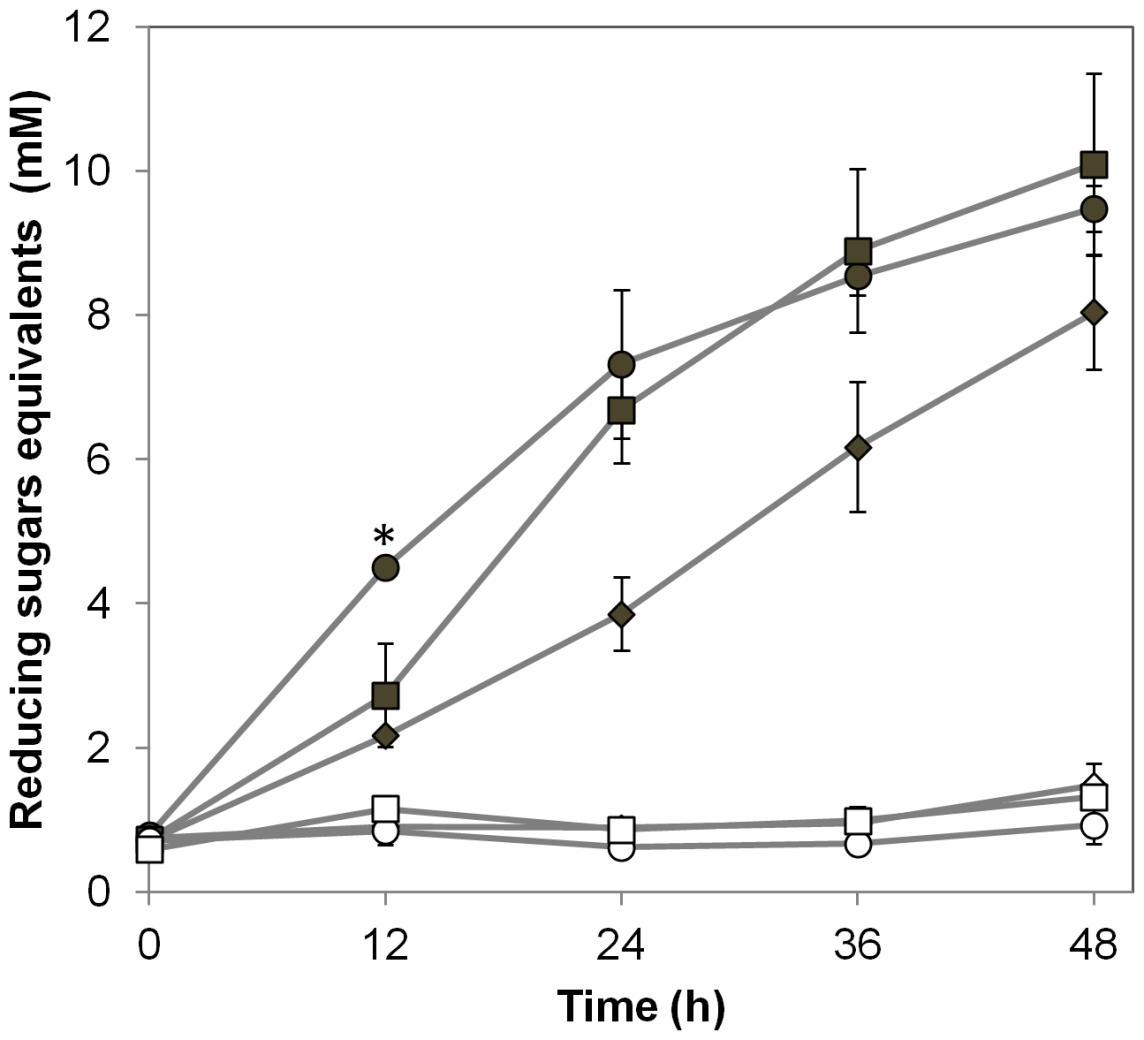
Hydrolytic enzymes (cellulase and xylanase) activity is not influenced by the presence of PcExl1. Endoglucanase from *T. reesei* (open symbols) was incubated with: buffer only (open diamonds); 150 µg/ml PcExl1 (open circles); and 150 µg/ml BSA (open squares). Endoxylanase from *T. logibrachiatum* was incubated with: buffer only (full diamonds); 150 µg/ml PcExl1 (full circles); and 150 µg/ml BSA (full squares). All reactions were carried out in 600 µl and agitated at 600 rpm at 30°C. Samples were taken every 12 h and reducing sugars were quantified by the DNS method, and compared to glucose or xylose standard curves. Reducing sugars concentration is expressed as glucose or xylose equivalents for reactions with cellulase or xylanase, respectively. Results are the arithmetic mean and standard deviations of three independent experiments. *Significant difference between reactions containing Exl1+ xylanase compared to the reactions containing BSA+xylanase, at each time point (*p*<0.05, Fisher’s LSD test).

Finally, because of opposing results in the literature on the existence of synergism between bacterial expansins and cellulases and/or xylanases [6], [14], [26], we looked for synergism between PcExl1 and these enzymes. Reactions with 0.25 U of endocellulase from *T. reesei* failed to produce reducing sugars with 2.5 mg Avicel as a substrate even at long incubation times, either in the presence or absence of PcExl1 (Figure 6), whereas the same amount of enzyme produced 2.5 mM of glucose equivalents using carboxymethyl cellulose in three hours (not shown), indicating that the enzyme was active. On the other hand, following incubation of xylan from beechwood with a xylanase from *T. longibrachiatum*, increased reducing sugars production through time reached 8 mM xylose equivalents at 48 h. Inclusion of PcExl1 resulted in a further increase in reducing sugars throughout the time, being significantly different only at 12 h of incubation. The effect of the protein at later times seems to be an unspecific consequence of the protein concentration in the reaction mixture, as the same concentration of the irrelevant protein BSA resulted in similar increments (Figure 6). Taking these results together, we conclude that no synergism between hydrolytic enzymes and PcExl1 occurs under these conditions, and that only at 12 h a modest increment of reducing sugars production can be obtained.

## Discussion

*Pectobacterium carotovorum* (formerly *Erwinia carotovora*), is a plant pathogen with important economic impact. We have studied an expansin-like gene (*exl*1) from an environmental strain of *P. carotovorum* isolated from an infected *Opuntia*. Biochemical analyses confirmed that PcExl1 binds and acts in a similar manner to BsEXLX1 from *B. subtilis* towards cellulose substrates. This is to be expected as the 3D model of PcExl1 predicts a carbohydrate-binding surface highly similar between the two proteins. Accordingly, binding parameters to Avicel and weakening of filter paper strips were indistinguishable between PcExl1 and BsEXLX1. This interaction with cellulose includes an ionic component revealed by increasing the ionic strength that favoured binding of PcExl1 but impaired binding of BsEXLX1. Very high concentrations of NaCl, however, seem to reverse the effect for BsEXLX1 as observed by Kim, et al. [20] that reported a binding increment from 250 mM to 1 M NaCl. Interaction of each expansin with plant cell walls was also different. BsEXLX1 binds preferentially to whole cell walls from wheat coleoptiles, and removal of pectin and hemicellulose reduces binding to the remaining cell wall fraction (mainly composed of cellulose) [10]. The opposite result was found in PcExl1, as pectin and hemicellulose seem to interfere with binding to cellulose. An explanation for this behaviour could be attributed to the different electrostatic nature of PcExl1 and BsEXLX1. Interaction of BsEXLX1 with acidic pectin and hemicellulose is favoured by the basic character of the protein, whereas negatively-charged PcExl1 could be repelled by these polysaccharides. This compares to another acidic protein from *Hahella chejuensis* that, even when it has been shown to bind to cell wall polysaccharides, has a preference for crystalline cellulose [27]. Consequently, pH influenced binding of PcExl1 WT and mutant variants to cell walls, resulting in unspecific binding at pH 3.5, and it decreased as the pH increased, causing the elimination of binding at pH 9, while BsEXLX1 showed binding of more than 50% in all conditions. It is interesting that the D82A mutant showed greater binding levels at all pHs compared to wild type PcExl1, suggesting that an excess of negative charge is impairing binding.

If cellulose is the main target of bacterial expansins, it remains an interesting question as to why expansins display different characteristics outside the PPBS. Analysis of the infection capabilities of *P. carotovorum* 101 strain showed that dicot plants are susceptible whereas monocots are resistant to disease. However, for most of the same plant species, BsEXLX1 presented higher binding levels to whole cell walls (up to 16-fold greater than PcExl1), and yet *B. subtilis* is not a known plant pathogen. Nevertheless, binding to the cell wall matrix is not a prerequisite for activity, as BsEXLX1 D2 mutants that replace Arg or Lys by Gln (R173Q/K180Q/K183Q and K145Q/K171Q/K188Q) lost their ability to bind to wheat coleoptile cell walls but developed increased cell wall creep activities [10]. Also, an expansin-like protein, cerato platanin, from the pathogen fungus *Ceratocystis platani*, disrupts Avicel and cotton fibres without stable binding in *in vitro* assays [28]. To date, there is no data regarding the required degree of bond stability between an expansin and its substrates in order to achieve the best activity. Differences between PcExl1 and BsEXLX1 could be related to the mechanism of interaction of each microorganism, *P. carotovorum* or *B. subtilis*, and its plant host. Local pH of the infected site by *P. carotovorum* is lightly alkaline [29], and at this condition significant differences on binding between PcExl1 and BsEXLX1 exist. Tissue maceration by *P. carotovorum* occurs by the action of extracellular enzymes, most importantly pectinases that are highly expressed early during infection. Expansins from *P. carotovorum* or *B. subtilis* would encounter cell wall matrices of differing compositions, and so each of these proteins would be better adapted to their particular environment (pH, ionic strength, etc). Experiments to determine the physiological role of expansin PcExl1 in our *P. carotovorum* 101 strain are being carried out to confirm this hypothesis.

Finally, lignocellulose is a highly abundant feedstock for the production of ethanol, and much research effort has been, and continues to be, directed towards finding enzymes able to overcome its recalcitrance to improve the yield of saccharification processes. Because expansins act on cellulose to loosen plant cell walls, they have been seen as a potential additive alongside hydrolytic activities to make processes more efficient. Indeed, reports indicate that production of reducing sugars by either cellulases or xylanases in reactions containing expansins from *B. subtilis* [26], [30] or *H. chejuensis* [14], [27] is increased. This is not the case for PcExl1, that even at very high concentration or long incubation time (640 µg/ml; 96 h, data not shown) did not generate an increase in the production of reducing sugars in combination with a cellulase, and only a modest increment was observed at early times of incubation times in combination with a xylanase. This is in agreement with results observed by Georgelis, et al. [6] in which a number of expansins from different plant pathogens failed to act in synergy with hydrolytic enzymes. Protein engineering of expansins, however, could be attempted in order to obtain more active variants. This would imply devising high-throughput methods that, in combination with an endocellulase, allow screening millions of mutant proteins.
